# Study on the Recycling of Phosphate Ore Waste Rock and Its Impact on Mortar Properties

**DOI:** 10.3390/ma19081568

**Published:** 2026-04-14

**Authors:** Ridong Fan, Baiyang Mao

**Affiliations:** 1 School of Environmental Science and Engineering, Donghua University, Shanghai 201620, China; 2 Shanghai Research Institute of Building Sciences Co., Ltd., Shanghai 200032, China; m19702658741@163.com

**Keywords:** phosphate waste rock, recycled sand, cement mortar, circular economy

## Abstract

To promote the resource recovery of phosphate mine tailings and alleviate the pressure caused by the growing scarcity of river sand, this study employs a research methodology combining macroscopic performance analysis with microscopic testing to systematically investigate the effects of three types of recycled sand containing varying proportions of phosphate mine tailings (flint (FS), phosphorite flint (PFS) and dolomitic limestone (DLS)) on the performance of mortar. The study focused on assessing the impact of recycled sand on the workability of mortar, water absorption, mechanical properties, pore structure, cement hydration characteristics, and environmental safety, and conducted a comprehensive evaluation of the project’s feasibility in conjunction with a cost analysis. The effect of DLS was most pronounced in terms of setting time. Water absorption tests show that when the proportions of FS, PFS, and DLS are all 25%, the mortar’s water absorption reaches its minimum value. In terms of mechanical properties, DLS showed a more pronounced increase in early-stage flexural strength, whilst PFS and FS demonstrated a more significant increase in later-stage strength. In terms of compressive strength improvement, PFS outperformed both FS and DLS. XRD and TG-DTA test results show that the three kinds of recycled sand have no adverse effect on cement hydration. SEM and MIP results confirmed that compared with river sand, the porosity of mortar mixed with FS was smaller and the pore structure was denser. Environmental safety assessments have shown that the heavy metal leaching concentrations in the mortar made from the three types of recycled sand are all significantly below the national limits, indicating good environmental compatibility. An economic analysis indicates that the “25% river sand + 75% FS” alternative offers the best economic benefits, resulting in cost savings of 93.27 CNY per cubic metre. In summary, the use of recycled sand derived from phosphate ore tailings as a substitute for river sand in the preparation of mortar is feasible from technical, environmental, and economic perspectives. This approach facilitates the recovery of solid waste resources, conserves natural resources, reduces the environmental burden, and promotes cost optimisation.

## 1. Introduction

The mining industry, as a globally vital foundational sector, inevitably generates substantial quantities of solid waste such as waste rock and tailings during mineral extraction and processing. The disorderly disposal of such waste has become a global ecological and environmental challenge [1]. Statistics indicate that global mining activities generate between 20 and 25 billion tonnes of solid waste annually. This waste not only occupies vast tracts of land resources but also disrupts surface landscapes, alters topography, and encroaches upon arable land, inflicting profound and enduring detrimental effects upon regional ecosystems and cultural environments [2,3,4]. Therefore, exploring pathways for the resource utilisation of mining waste to achieve ecological recycling and enhance economic value by transforming waste into treasure has become a critical issue in the fields of sustainable mining development and environmental protection.

In recent years, with the increasing application of phosphate rock in agricultural fertilisers, pharmaceuticals, and other sectors, market demand for phosphate raw materials has continued to rise. China’s annual phosphate rock production has now reached approximately 900,000 tonnes [5,6]. During open-pit phosphate mining operations, the substantial quantities of unusable interlayers (flint limestone, limestone, marlstone, etc.) and overburden (topsoil, clay, marlstone, etc.) generated following blasting activities form vast quantities of phosphate waste rock [7,8]. Although such waste rock exhibits relatively stable chemical properties, its prolonged stockpiling exerts significant pressure on the ecological environment. Not only does it compromise the integrity of the landscape, but it may also pose potential environmental risks through processes such as leaching by rainwater. Moreover, its potential resource value remains largely untapped [9,10,11]. It is noteworthy that the chemical composition and physical properties of phosphorite waste rock bear a high degree of similarity to the alkaline untreated clay, sand, and aggregates commonly employed in civil engineering. This provides a crucial material basis for its utilisation as an alternative resource in construction materials [12,13]. Concurrently, the rapid expansion of the construction industry and accelerated urbanisation have led to a sharp increase in market demand for natural building resources such as river sand and aggregates. The global annual extraction of natural sand and gravel for construction purposes has now reached approximately 25 billion tonnes [14]. River sand, as the core component of mortar, constitutes approximately 75% of its total volume. However, its excessive extraction has led to a series of environmental issues, including river ecological damage and channel siltation, resulting in the increasingly depleted natural river sand resources [15,16]. The production process of manufactured sand, however, faces the dual challenges of resource depletion and environmental disturbance [17]. To alleviate this contradiction, academia has explored substituting river sand with industrial by-products such as ceramic waste, brick rubble, glass scrap, and iron tailings sand for mortar preparation. Some studies have demonstrated that incorporating small quantities of tailings can effectively enhance mortar strength and durability [18,19,20]. However, existing research has yet to address the feasibility of substituting natural river sand in mortar with recycled sand from phosphorite waste rock, thereby failing to fully realise the resource potential of phosphorite waste rock. Processing phosphorite waste rock into recycled sand to replace river sand in mortar not only alleviates the pressure on natural river sand extraction and mitigates the crisis of natural resource depletion, but also achieves the reduction and resource utilisation of phosphorite waste rock. Minimising its adverse impact on the ecological environment at source aligns with the development principles of “ecological circulation” and “green building”.

Based on the above research background and existing research gaps, this paper specifically carries out the following work: Three types of waste rocks, flint, phosphorite flint, and dolomitic limestone, produced in the process of phosphate mining, are selected and prepared into recycled sand to replace river sand for mortar preparation. The effects of three kinds of recycled sand with different contents on the physical properties, water absorption, and mechanical properties of fresh mortar were systematically tested and analyzed. The environmental safety of recycled sand mortar was evaluated by a leaching toxicity test to ensure that it meets the requirements of environmental protection. At the same time, the cost analysis is carried out to clarify the economic benefits of different reclaimed sand alternatives. In addition, the effects of recycled sand on the hydration characteristics of cement and the pore structure of mortar were investigated by means of XRD, TG-DTA, SEM, and MIP. The feasibility of replacing river sand with recycled sand of phosphate rock waste was comprehensively demonstrated, which provided data support and practical guidance for its large-scale engineering application.

## 2. Materials and Methods

### 2.1. Materials

The river sand used in this study was purchased from Gansu Shunxiang New Energy-saving Building Materials Co., Ltd. (Lanzhou, China). Flint (FS), phosphorite flint (PFS), and dolomite limestone (DLS) were all sourced from Gansu Jingtieshan Mining Co., Ltd. (Zhangye, China); their specific chemical compositions are shown in Table 1, and their physical properties are shown in Table 2. The cement was purchased from Pingliang Conch Cement Co., Ltd. (Pingliang, China); its chemical composition is shown in Table 3.

### 2.2. Methods

This study employed three types of recycled sand produced from phosphorite waste rock processing as a substitute for river sand in mortar formulations. The replacement ratios of recycled sand for river sand were set at 0%, 25%, 50%, 75%, and 100%. In the preliminary stage of the experiment, both river sand (reference sand) and recycled sand were dried in an oven at 100 ± 10 °C for 24 h to eliminate interference from surface-adsorbed water on the experimental results. The experimental water-cement ratio was set at 0.5, with cement fixed at 450 g and aggregate total at 1350 g. For the five replacement ratios, the recycled sand additions were 0 g, 337.5 g, 675 g, 1012.5 g, and 1350 g, respectively, while water content was uniformly set at 300 g.

#### 2.2.1. Slump Test

The slump of mortar was measured according to the Chinese standard GB/T 2419-2005 [21]. Uniform fresh mortar was mixed according to the proportion specified in the standard to ensure that the mortar was not agglomerated and had good workability. The mortar was filled into the mold to test the slump of the mortar. Three parallel tests were performed in each group, and the average value was taken as the final result.

#### 2.2.2. Setting Time

The initial and final setting times of the cement mortar were determined using a Vicat apparatus in accordance with the Chinese national standard GB/T 1346-2011 [22]. The test environment was maintained at a constant temperature of 20 ± 1 °C and a relative humidity (RH) of ≥90%.

#### 2.2.3. Water Absorption Performance Test

The water absorption performance of the mortar was tested according to the Chinese standard JGJ/T 70-2009 [23]. During the test, the sample was cured for 28 d. After the curing was completed, the sample was placed at 105 ± 5 °C for 48 h, and the drying mass *m*_0_ was weighed. After that, it was soaked in water again for 48 h. The excess water on the surface was removed with a wet towel, and the mass *m*_1_ after water absorption was weighed, and the water absorption rate *m*_w_ was calculated according to Equation (1).

(1) $$m_{w}=\frac{m_{1}-m_{0}}{m_{0}}\times 100\%$$

#### 2.2.4. Mechanical Properties Testing

Specimens with dimensions of 40 mm × 40 mm × 160 mm were fabricated following GB/T 17671-2021 [24]. The specimens were cured in a standard curing chamber at 20 °C with RH ≥ 95% for curing ages of 28 d, respectively. Flexural and compressive strengths were tested using a microcomputer-controlled universal testing machine (Jinan Huayue Testing Machine Co., Ltd, Jinan, China) at a loading rate of 2.4 kN/s. Each test group included three parallel specimens, and the average value was adopted as the final result.

#### 2.2.5. Microscopic Test

Testing was conducted using a Shimadzu XRD-7000 X-ray diffractometer (Shimadzu Corporation, Kyoto, Japan) under the following conditions: Cu target (Kα radiation), tube voltage 40 kV, tube current 30 mA, scanning range 7–60°, scanning rate 5°/min, step size 0.02°. The XRD patterns were analysed to determine the types of hydration products and changes in characteristic peak intensities. Thermal stability testing of mortar was conducted using an STA449 F3 thermogravimetric analyser (NETZSCH Analyzing & Testing, Selb, Germany)under the following conditions: nitrogen atmosphere, gas flow rate 50 mL/min, heating range 20–800 °C, heating rate 10 °C/min. Observe the microstructure of the mortar using an Apreo 2 field emission scanning electron microscope (Thermo Fisher Scientific, Waltham, MA, USA).

#### 2.2.6. Porosity Test

The porosity of cement mortar cured for 28 d was determined using an Autopore IV 9520 mercury porosimeter (Micromeritics Instrument Corporation, Norcross, GA, USA). The pore diameters penetrated by mercury at each pressure were calculated via the Washburn equation. The high-pressure range is 140–420 kPa, and the low-pressure range is 1.5–350 kPa. Before testing, fragments with diameters ranging from 3 to 6 mm were immersed in isopropanol for 24 h and dried in a vacuum oven at 40 °C for 12 h.

#### 2.2.7. Hydration Heat Test

An eight-channel TAM AIR isothermal microcalorimeter (TA Instruments, New Castle, DE, USA) was employed to continuously monitor the hydration heat release rate for 72 h at a constant temperature of 20 ± 0.02 °C. The effect of recycled sand with different types on the hydration kinetics of the cementitious system was investigated.

#### 2.2.8. Heavy Metal Ion Concentration Testing

Take mortar specimens with 100% recycled sand replacement and cured for 28 d. After crushing, grind and sieve through a 100-mesh standard sieve. Accurately weigh 100 g of the sample and place it in a 2 L polyethylene leaching bottle. Add deionised water at a liquid-to-solid ratio of 10:1 (L/kg), then seal the leaching bottle. Place the leaching flasks on a horizontal shaker and agitate for 8 h at (23 ± 2) °C with a shaking frequency of 110 ± 10 cycles per minute. After standing for 16 h, filter using medium-speed qualitative filter paper (Membrane Solutions, Auburn, WA, USA) and collect the filtrate. The filtrate was then subjected to secondary filtration using a 0.45 μm filter membrane to obtain the leachate. The concentrations of heavy metal ions, including Ag, As, Ba, Cd, Cr, Hg, Pb, and Zn in the leachate were determined using an inductively coupled plasma optical emission spectrometer (ICP-OES, Thermo Scientific, Waltham, MA, USA). Three parallel samples were tested per group, with the mean value recorded. The concentrations of heavy metal ions in the filtrate from mortar prepared using phosphorite waste rock were also measured.

## 3. Results and Discussion

### 3.1. Mortar Slump and Initial Setting Time

**Figure 1:**
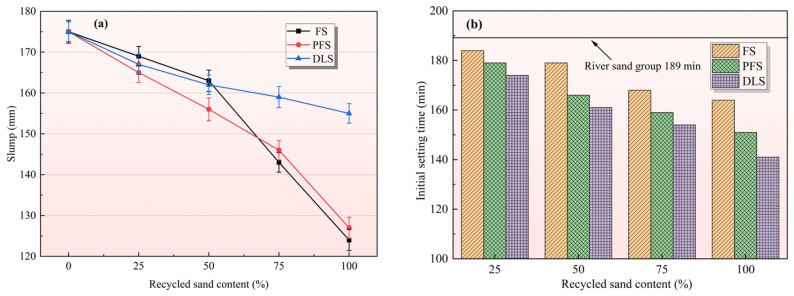
Effect of recycled sand replacement ratio on mortar properties: (**a**) slump, (**b**) setting time.

The relationship between the recycled sand replacement ratio and mortar slump is illustrated in Figure 1a. As the replacement ratio of recycled sand (FS, PFS, and DLS) increases from 0% to 100%, the mortar slump consistently decreases. This indicates that the incorporation of recycled sand significantly reduces the workability of freshly mixed mortar, with varying degrees of impact observed across different types of recycled sand. The slump of the control group (100% river sand) was 175.3 mm. When replacing 25%, 50%, 75%, and 100% of the sand with FS recycled sand, the slump decreased successively to 169.2 mm, 163.6 mm, 143.1 mm, and 124.5 mm, with the reduction becoming progressively greater. At 100% replacement, it reduced emissions by 28.98% compared to the control group, representing the most significant impact among the three types of recycled sand. Under the specified replacement ratios for PFS recycled sand, the slump values were 165.4 mm, 156.2 mm, 146.2 mm, and 127.7 mm, respectively. At 100% replacement, the slump reduction was 27.15%, slightly lower than that of the FS group. The DLS group exhibited the most gradual decline in slump, with values of 167.1 mm, 162.3 mm, 159.2 mm, and 155.8 mm at each replacement level. At 100% replacement, the reduction was merely 11.12%, demonstrating optimal retention of workability.

The core reason for this phenomenon is directly related to the physical properties of recycled sand: on the one hand, the water absorption rates of the three types of recycled sand increased by 116%, 134%, and 167% respectively compared to river sand (Table 2). The high water absorption rate causes recycled sand to adsorb a significant amount of free water during mixing, resulting in a reduction in the effective water content in the mortar paste and a decrease in its workability [25].

Figure 1b illustrates the effect of recycled sand replacement ratio on the initial setting time of mortar. As the proportion of recycled sand increases in the diagram, the initial setting time of the mortar continues to decrease, with DLS exhibiting the most pronounced acceleration effect on the setting process. The initial setting time for the control group (100% river sand) was 189 min. When replacing 25%, 50%, 75%, and 100% of the sand with DLS recycled sand, the initial setting times were 174 min, 161 min, 154 min, and 141 min, respectively. Under the specified replacement ratio for PFS recycled sand, the initial setting times were 179 min, 166 min, 159 min, and 151 min, respectively. The initial setting time of FS recycled sand exhibited a relatively moderate decrease, with values of 184 min, 179 min, 168 min, and 164 min at different replacement levels. Analysis indicates that the core driver behind the shortened initial setting time remains the high water absorption rate of recycled sand. Recycled sand rapidly adsorbs free water from the slurry after mixing, thereby reducing the moisture content in the hydration environment surrounding cement particles. This accelerates the hydration reaction rate, leading to the premature hardening and drying of the mortar. The acceleration effect of DLS on initial setting time is most pronounced, and is related not only to high water absorption but also to its physical properties. The fine particle content of DLS (26.85%) is significantly higher than that of river sand (10.30%) (Table 2). The abundance of fine particles provides additional crystallisation sites for cement hydration. Furthermore, its pH value (9.17) is slightly higher than that of other sand samples; this weakly alkaline environment may further promote the initiation of hydration reactions [26].

### 3.2. Water Absorption Properties of Mortar

**Figure 2:**
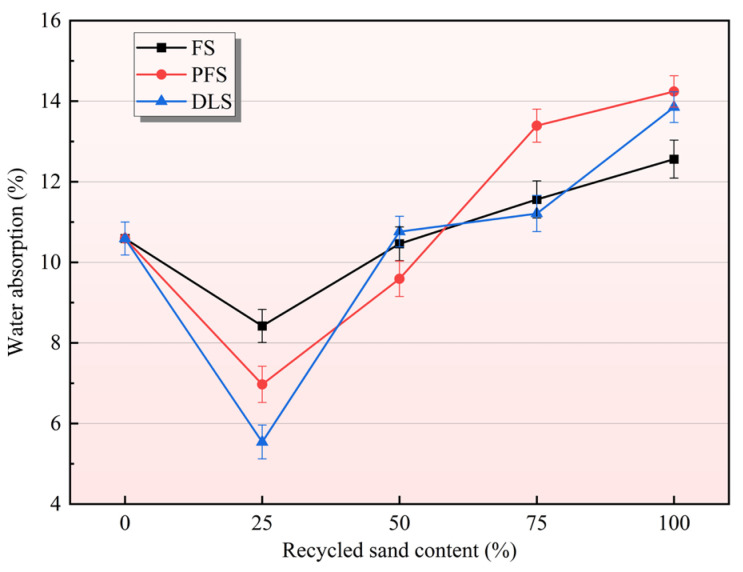
Effect of Recycled Sand Replacement Ratio on Mortar Water Absorption Properties.

The relationship between the recycled sand replacement ratio and mortar water absorption behaviour is illustrated in Figure 2. As the replacement ratio of recycled sand (FS, PFS, and DLS) increases from 0% to 100%, the water absorption rate of the mortar exhibits a characteristic pattern of first decreasing and then increasing. Moreover, a 25% replacement ratio represents the optimal blending proportion, at which point water absorption performance is at its optimum. The degree of influence varies significantly between different types of recycled sand. The reference group (100% river sand) exhibited a water absorption rate of 10.59%. When the replacement rate of recycled sand reached 25%, all three types of recycled sand reduced the mortar’s water absorption rate to its minimum value: The FS group recorded 8.42% (a reduction of 20.49% compared to the control group), the PFS group 6.97% (a reduction of 34.18%), and the DLS group 5.54% (a reduction of 47.69%). Among these, DLS demonstrated the most pronounced optimisation effect, achieving a reduction of nearly half, followed by PFS, while FS exhibited a relatively moderate decrease. The core reason for this phenomenon is directly related to the optimisation of the internal structure of the mortar. At a low replacement rate of 25%, the fine particles in recycled sand (particularly DLS fine particles, which accounted for 26.85%—significantly higher than the 10.30% in river sand) exerted an efficient filling effect. They filled the voids between river sand particles, enabling the mortar to form a densely packed structure. This markedly reduced internal porosity and minimised water penetration pathways, thereby suppressing water absorption capacity [27]. Concurrently, the coarse surface texture and high water absorption rate of recycled sand (116%, 134%, and 167% higher than river sand, respectively) enable it to adsorb free water during mixing. This reduces capillary voids within the paste, further enhancing its water-repellent properties.

When the proportion of recycled sand exceeds 25%, the water absorption rate begins to rise steadily. At 50% replacement, the water absorption rates for the FS, PFS, and DLS groups were 10.46%, 9.59%, and 10.76%, respectively, approaching or slightly exceeding those of the control group. At 75% replacement, the values further increased to 11.56%, 13.39%, and 11.21%. At 100% replacement, the values reached their peak at 12.59%, 14.24%, and 13.85%, respectively, representing increases of 18.89%, 34.47%, and 30.78% compared to the control group, with the PFS group showing the greatest improvement. When the replacement ratio is excessively high, particles readily form interlocking and entangled structures, preventing close packing and instead increasing the internal porosity of the mortar. Concurrently, the high water absorption of excessive recycled sand leads to insufficient slurry moisture, resulting in incomplete cement hydration. This causes the interface transition zone (ITZ) to become loose, forming more interconnected pores that provide convenient pathways for water penetration. Ultimately, this leads to a significant increase in water absorption [28].

The variation in water absorption properties among different recycled sands fundamentally stems from differences in their physical characteristics. DLS exhibits the highest content of fine particles and demonstrates the strongest filling effect at low replacement levels, hence achieving the greatest reduction in water absorption rate. PFS particles exhibit greater angularity, resulting in more pronounced voids formed during high-overlap installation; consequently, they demonstrate the greatest increase in water absorption rate. FS exhibits a slender, lamellar structure, with intergranular interlocking resistance falling between the two extremes. Consequently, the variation in water absorption capacity is relatively gradual. This pattern resonates with subsequent analyses of mechanical properties and pore structure, collectively corroborating the underlying mechanism linking recycled sand replacement ratios to the internal structure and macroscopic performance of mortar.

### 3.3. Mechanical Properties of Mortar

**Figure 3:**
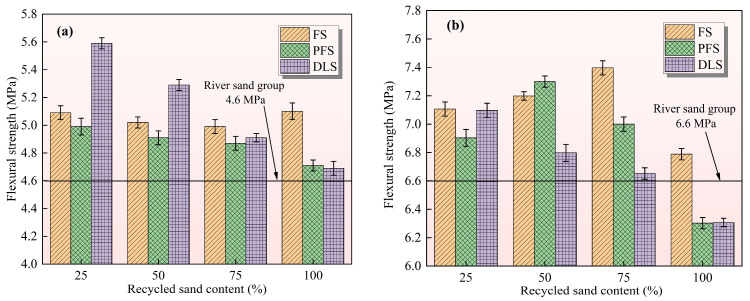
Effect of recycled sand replacement ratio on mortar flexural strength: (**a**) 7 days, (**b**) 28 days.

Flexural strength is the core indicator characterising mortar’s toughness and crack-resistance capability. Figure 3 illustrates the relationship between the recycled sand replacement ratio and the flexural strength of mortar at 7 d (early stage) and 28 d (late stage). The overall trend indicates that all three types of recycled sand possess the potential to enhance the flexural strength of mortar, though significant differences exist across different curing periods. DLS is more conducive to enhancing early strength development, whereas FS and PFS demonstrate superior performance in later stages. This distinction is directly attributable to the physical properties of recycled sand, its hydration activity, and the underlying microstructural mechanisms, which align with subsequent hydration characteristics and conclusions drawn from pore structure analysis.

The control group (100% river sand) exhibited a 7-day flexural strength of 4.60 MPa, with significant variations observed across different recycled sand types. The DLS exhibits its strongest enhancement effect in the early stages, with its intensity following a pattern of “initial increase followed by decrease” as the replacement ratio increases. At a 25% replacement ratio, it reaches a peak value of 5.59 MPa (representing a 21.52% increase), and even at 100% replacement, it remains slightly higher than the control group. The core reason lies in its significantly higher fine particle content (26.85%) compared to river sand. At low replacement ratios, it fills voids to form a compact structure. Furthermore, its high water absorption rate (167% greater than river sand) accelerates early cement hydration, resulting in denser interfacial bonding. At high replacement levels, excessive fine particles increase the water demand of the slurry, leading to a loose interfacial transition zone (ITZ) and reduced strength [29]. The flexural strength exhibits a gradual variation with replacement ratio, remaining stable at approximately 5.0 MPa across all ratios (representing an increase of 8.5% to 10.9%), with fluctuations limited to 2.2%. Benefiting from its coarse, elongated flake structure, the strong interlocking between particles hinders early crack propagation; however, the low content of fine particles (7.07%) and the delayed development of pozzolanic activity result in a moderate increase in early strength. The PFS strength decreased slightly with replacement levels, reaching 4.99 MPa at 25% replacement (an 8.5% increase) and dropping to 4.71 MPa at 100% replacement (a 2.4% increase). The dissolution of apatite releases PO_4_^3−^ ions, which inhibit the early hydration of cement. This results in insufficient formation of hydration products, leading to weakened interfacial bonding and preventing the full exploitation of the advantages offered by the angular edges of the particles [30].

The control group exhibited a flexural strength of 6.60 MPa at 28 days. As the curing age increased, the strengthening effect of the three types of recycled sand became more pronounced, with the optimum replacement ratio and its pattern changing. FS exhibited the most pronounced late-stage enhancement, with strength progressively increasing with replacement volume. Peak strength of 7.39 MPa (an 11.97% increase) was attained at 75% replacement, remaining stable even at 100% replacement. Owing to the weak pozzolanic activity of its microcrystalline quartz, it reacts with Ca(OH)_2_ in later stages to form additional C-S-H gel, densifying pores and interfaces [31]. Simultaneously, the overlapping of the laminar structure enhances stability and increases resistance to crack propagation [32]. PFS strength exhibits a “rise-then-stabilise” pattern, with the 50% replacement ratio reaching a peak value of 7.29 MPa (representing a 12.12% increase, the highest subsequent rise). Strength stabilises between 7.15 and 7.29 MPa across replacement ratios from 50% to 100%. At a 50% replacement ratio, the angular edges of the particles form a stable “skeletal support”, which both impedes crack propagation and prevents excessive porosity [33]. The later-stage hydration inhibition effect diminishes, allowing the hydration products to fill the interfacial voids, thereby significantly enhancing the bond strength. The optimal replacement volume for DLS remains at 25%, with a peak pressure of 7.10 MPa (an increase of 7.58%), though the rate of improvement is less pronounced than in earlier stages. Due to its enhancement mechanism being primarily based on “physical packing”, no significant secondary hydration reaction occurs in the later stages. Consequently, interfacial defects at high substitution levels cannot be compensated for by subsequent hydration.

The mechanism by which recycled sand enhances the flexural strength of mortar can be summarised as follows: Early-stage performance relies on the filling effect of fine particles and interfacial densification (where DLS demonstrates significant advantages), whilst later-stage performance depends on pozzolanic secondary reactions and crack-resistant particle morphology (where FS and PFS show marked advantages). This provides targeted guidance for mortar mix design tailored to the requirements of different curing stages.

**Figure 4:**
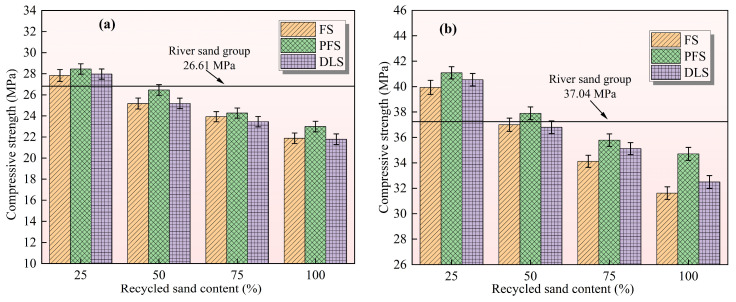
Effect of recycled sand replacement ratio on mortar compressive strength: (**a**) 7 days, (**b**) 28 days.

Compressive strength is the core mechanical indicator for measuring the load-bearing capacity of mortar. Figure 4 illustrates the relationship between recycled sand replacement ratio and the compressive strength of mortar at 7 d and 28 d. The overall trend indicates that all three types of recycled sand can enhance the compressive strength of mortar at specific replacement levels, with the optimum replacement level being 25% for each. The enhancement effect of PFS is most pronounced, followed by FS and DLS. When the replacement ratio exceeds 25%, the compressive strength of all three types of recycled sand exhibits a declining trend. The control group (100% river sand) exhibited a 7 d compressive strength of 26.61 MPa, serving as the benchmark for early-stage load-bearing performance. The influence characteristics of different recycled sands were highly consistent. Only when the replacement ratio of recycled sand reaches 25% do all three types of recycled sand yield compressive strengths exceeding those of the control group. The FS group recorded 27.83 MPa (an increase of 4.57%), the PFS group 28.45 MPa (an increase of 6.91%), and the DLS group 27.97 MPa (an increase of 5.11%).

Among these, PFS demonstrated the most favourable early-stage strengthening effect, closely linked to its physical properties. The fine particle content of PFS (11.00%) is comparable to that of river sand. At low replacement ratios, fine particles can fill internal voids within the mortar while optimising granular packing density. Concurrently, their rough edges enhance mechanical interlocking with the cement paste, thereby increasing interfacial bond strength. The flake-like structure of FS and the high content of fine particles in DLS can still exert a certain degree of filling effect even at low replacement levels, thereby modestly enhancing strength. When the replacement ratio of recycled sand exceeds 25%, the compressive strength of all three types of recycled sand falls below that of the control group, and continues to decrease as the replacement ratio increases. At replacement levels of 50% to 100%, the strength of the FS group decreased from 26.32 MPa to 24.15 MPa, the PFS group from 25.98 MPa to 23.87 MPa, and the DLS group from 26.11 MPa to 22.93 MPa. The core cause of this phenomenon lies in structural deficiencies under high replacement volumes: The elongated, flake-like particles of FS readily overlap and intertwine, forming numerous interconnected voids that reduce the slurry’s density. DLS exhibits low hardness and excessive fine particles; the surplus of fine particles increases the water demand of the slurry, resulting in a loose interface transition zone (ITZ) that cannot withstand early-stage loads. Whilst PFS exhibits outstanding angular particle advantages, the voids formed by particle interlocking at high replacement levels cannot be adequately filled by hydration products, thereby negating the strength-enhancing effect.

The control group exhibited a compressive strength of 37.04 MPa at 28 d. As the curing age increased, the strengthening effect of recycled sand became more pronounced, yet the optimum replacement ratio remained at 25%, with the strength difference becoming more significant. At a replacement ratio of 25%, all three types of recycled sand achieved their peak compressive strength, with the increase being greater than that observed in the earlier stages. The FS group recorded 39.94 MPa (an increase of 7.83%), the PFS group 41.08 MPa (an increase of 10.91%, exhibiting the most pronounced enhancement effect), and the DLS group 40.54 MPa (an increase of 9.45%). This outcome stems from the synergistic effect of enhanced late-stage hydration and particle interactions. PFS particles exhibit greater angularity, resulting in enhanced mechanical interlocking with the cement paste. Their moderate fine particle content enables both pore filling for compact packing and provision of ample crystallisation sites for hydration reactions, yielding a denser interfacial transition zone. The weak pozzolanic activity of FS microcrystalline quartz reacts with Ca(OH)_2_ generated during cement hydration, forming additional C-S-H gel. This further fills pores and densifies the structure, partially compensating for early-stage defects [34]. The filling effect of DLS’s highly fine particles continues to exert its influence in the later stages, with more robust interfacial bonding resulting in a greater increase in strength compared to the initial phase. When the replacement ratio exceeds 25%, the later-stage compressive strength also exhibits a declining trend, with the rate of decrease consistent with that observed in the early stages. At replacement levels of 75% to 100%, the strength of the FS group decreased from 36.52 MPa to 34.87 MPa, the PFS group from 35.98 MPa to 33.76 MPa, and the DLS group from 35.11 MPa to 32.45 MPa, all falling below that of the control group. This is because structural defects under high replacement ratios cannot be fully compensated for by subsequent hydration, as the volume of pores formed by overlapping FS flake particles exceeds the filling capacity of the pozzolanic reaction products [35]. The characteristic of DLS’s relatively low hardness becomes more pronounced under subsequent loading, rendering it incapable of effectively transmitting stress [36]. Although PFS is fully hydrated, the voids between particles still reduce the overall density of the slurry, leading to a decrease in compressive strength.

In summary, the mechanism by which recycled sand enhances mortar compressive strength can be summarised as follows: in the early stages, it relies on the filling effect of fine particles and the mechanical interlocking force of particle edges; in the later stages, this is supplemented by the interface densification effect of hydration products. Moreover, a 25% replacement ratio represents the optimal proportion for achieving synergistic optimisation of “filling, bonding and hydration”. PFS, owing to its abundant angular particles and moderate content of fine particles, demonstrates superior performance in both filling efficacy and interfacial bonding, thereby delivering the most effective reinforcement.

### 3.4. Hydration of Mortar Cement

**Figure 5:**
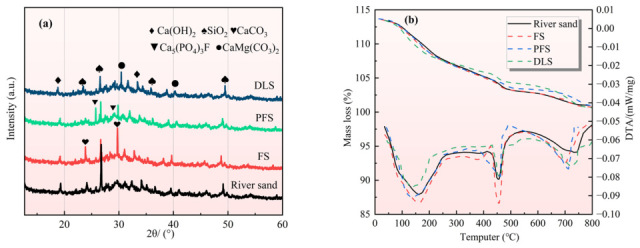
Effect of Different Recycled Sands on Cement Hydration: (**a**) XRD; (**b**) TG-DTA.

To precisely analyse the composition of mortar hydration products and mineral evolution characteristics, unhydrated sand particles were removed from samples before testing. XRD analysis was conducted on the 28 d cured reference group (100% river sand) and mortar samples, where three types of recycled sand fully replaced the sand content. The results are presented in Figure 5a. XRD patterns reveal that common hydration products such as Ca(OH)_2_, SiO_2_, and CaCO_3_ were detected in all mortar samples. Concurrently, characteristic new peaks appeared in the three categories of recycled sand groups, respectively. The CaCO_3_ peak was markedly enhanced in the FS group, while the PFS group exhibited a characteristic peak for Ca_5_(PO_4_)_3_F. The DLS group showed an additional peak for CaMg(CO_3_)_2_ alongside a concurrent intensification of the CaCO_3_ peak. This indicates that recycled sand does not inhibit the formation of hydration products but instead retains its original mineral composition, confirming its low reactivity as an aggregate and its lack of detrimental interference with cement hydration. It is noteworthy that characteristic peaks of cement clinker minerals such as C_3_S and C_2_S were not detected in any of the samples, indicating that the cement within the mortar matrix has undergone complete hydration [37].

Compared with the reference group, the SiO_2_ peaks in all three regenerated sand substitution groups exhibited a diminishing trend. The primary reason lies in the fact that the main mineral in the reference group’s river sand is quartz (SiO_2_). The core minerals of the three types of recycled sand are calcite (FS), apatite (PFS), and dolomite with calcite (DLS), with extremely low quartz content. The intensity of the characteristic SiO_2_ peak in the XRD pattern was significantly lower than that of the reference group, which is entirely consistent with the mineral composition analysis of the raw materials. The CaCO_3_ peak in mortar incorporating FS aggregate is markedly enhanced, owing to the core mineral of FS being calcite (CaCO_3_), whose content far exceeds the calcite impurities present in river sand. The Ca(OH)_2_ peak exhibits slight attenuation, attributed to the microcrystalline quartz within the fibre-reinforced cement composite possessing weak pozzolanic activity. This material reacts slowly with the Ca(OH)_2_ generated during cement hydration, producing minor quantities of C-S-H gel and thereby slightly depleting the Ca(OH)_2_ content [38]. Mortar incorporating PFS exhibits additional characteristic peaks for Ca_5_(PO_4_)_3_F, owing to the core mineral of PFS being fluorapatite (Ca_5_(PO_4_)_3_F). The Ca(OH)_2_ peak exhibits a slight reduction, attributable to the precipitation of trace calcium phosphate compounds formed by the combination of PO_2_^3−^ leached from PFS with Ca^2+^ released during cement hydration, concurrently consuming a portion of the Ca(OH)_2_ [30]. The CaMg(CO_3_)_2_ peak (a characteristic dolomite peak) in the DLS group exhibits synchronous enhancement with the CaCO_2_ peak, as dolomite constitutes the core mineral in DLS and frequently occurs in association with calcite. The Ca(OH)_2_ peak exhibits slight attenuation. This occurs partly because Mg^2+^ ions within dolomite react with hydration products to form trace amounts of hydrated micaceous compounds. Concurrently, carbonate minerals serve as highly efficient crystallisation nuclei for C-S-H gel, accelerating the deposition of hydration products and thereby indirectly consuming Ca(OH)_2_ [39].

To further elucidate the effects of recycled sand on the degree of cement hydration, the evolution of hydration products, and mineral reactions. TG-DTA testing was conducted on mortar specimens cured for 28 d, with a temperature range of 20–800 °C. The mass loss characteristics of TG curves directly reflect the decomposition patterns of different hydration products and the extent of mineral reactions, with DTA curves providing supplementary information for determining reaction types. The mass loss of TG in the figure can be divided into four core characteristic stages. The range of 30–150 °C represents the stage of removal of free water and bound water from hydration products. This stage primarily corresponds to the removal of interlayer water/adsorbed water from C-S-H gel, calcium aluminate hydrate (AFt), and monosulphate calcium aluminate (AFm) [40]. The mass loss rate at this stage directly reflects the total quantity of hydrated gel-like products and serves as a key indicator for evaluating the completeness of cement hydration. The sequence of mass loss rates at this stage is as follows: DLS > FS > river sand > PFS. This variation stems from the regulatory effect of recycled sand on cement hydration. The dual carbonate system (dolomite and calcite) in DLS exhibits a pronounced nucleation effect, significantly accelerating cement hydration. This promotes the extensive formation of hydration products such as C-S-H gel and AFt/AFm, leading to a corresponding increase in combined water content. Consequently, it exhibits the highest loss rate [41]. The calcite crystal nucleus effect in FS, combined with the weak pozzolanic activity of microcrystalline quartz, mildly promotes hydration. The yield of hydration gel products is slightly lower than that of DLS, with the second-lowest loss rate. River sand is inert quartz, exhibiting no accelerative or reactive properties. The hydration gel products are formed solely through the cement’s own hydration process, with a loss rate at the benchmark level. The PO_4_^3−^ dissolved by PFS forms a passivation film, which significantly inhibits the early hydration of cement. The amount of hydration gel products is the least, and the porosity of the slurry is high, the free water content is high, the proportion of free water removal is large, and the proportion of bound water removal is small [30], so the overall loss rate is the lowest.

The 400–500 °C range constitutes the decomposition stage of calcium hydroxide (CH), wherein CH decomposes at elevated temperatures into CaO and H_2_O. The loss rate during this phase directly correlates with the quantity of CH formed within the mortar [42]. Indirectly reflects the degree of cement hydration—the greater the amount of CH produced, the more complete the hydration. PFS mortar exhibited the most markedly low loss rate, owing to the retarding effect of PO_4_^3−^, resulting in the lowest degree of hydration and minimal CH formation. This represents the most direct manifestation of the retarding effect (consistent with the weakest Ca(OH)_2_ peak observed in XRD analysis). The calcite nucleation effect in FS mortar accelerates hydration, significantly increasing CH production. However, the pozzolanic activity of microcrystalline quartz consumes CH at a slower rate than its generation, ultimately resulting in a net CH production exceeding the baseline with the highest loss rate. The double carbonate nucleation effect in DLS mortar similarly promotes CH formation, but carbonate reacts with C_3_A in the cement to form calcium aluminate, thereby consuming large quantities of CH. Moreover, this reaction proceeds at a significantly faster rate than the pozzolanic reaction in FS, resulting in a net CH yield slightly below the baseline and a loss rate lower than that of river sand mortar (corresponding to the “slightly diminished Ca(OH)_2_ peak” observed in XRD analysis). The 600–800 °C range constitutes the carbonate decomposition stage: 600–700 °C sees thermal decomposition of calcite (CaCO_3_), while 700–800 °C involves secondary decomposition of dolomite (CaMg(CO_3_)_2_). Loss rates correlate closely with the aggregate’s inherent carbonate content and carbonate formation/reaction during the hydration process [43]. The quality loss at this stage primarily stems from the carbonate minerals inherent in the aggregates themselves, with the variation being extremely pronounced. DLS and FS mortars exhibit relatively high loss rates, with DLS aggregates containing substantial quantities of calcite and dolomite, and FS aggregates containing significant amounts of calcite. These two components constitute the primary sources of loss at this stage. Carbonates in river sand and PFS aggregates are present only as trace impurities, exhibiting no significant thermal decomposition reaction. This corresponds with the absence of pronounced CaCO_3_ characteristic peaks in the XRD analysis. After 800 °C, it is the residual mass stage [44], and the curve tends to be gentle. The residues are mainly unhydrated cement particles, aggregate inert minerals (quartz, apatite), and inorganic oxides (CaO, SiO_2_, etc.) after the decomposition of hydration products. Among them, apatite (Ca(PO_4_)_3_F) has strong thermal stability, and there is no obvious mass loss below 800 °C, which has become a unique sign of the TG curve of apatite rock mortar.

### 3.5. Mortar Hydration Heat

**Figure 6:**
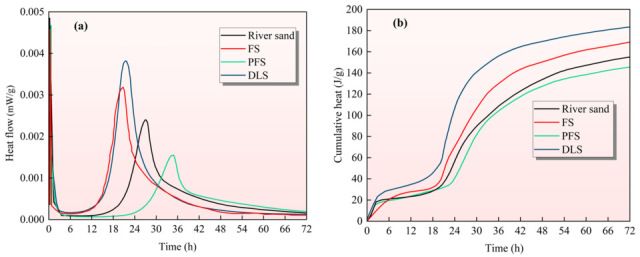
Effect of Different Recycled Sands on Cement Hydration Heat: (**a**) Heat Flux; (**b**) Cumulative Heat.

The hydration heat serves as a core indicator reflecting the cement hydration rate, reaction extent, and energy release characteristics. By examining the heat flux rate and cumulative heat release of mortar over 0–72 h (Figure 6), the distinct regulatory mechanisms of the three types of recycled sand on the cement hydration process can be precisely elucidated. The overall pattern indicates that the effect of recycled sand on hydration heat is fundamentally determined by its mineral composition. DLS and FS exhibit a setting-promoting effect, with DLS demonstrating a stronger setting-promoting action. PFS exhibits a retarding effect, a conclusion that fully corroborates the findings from the preceding XRD and TG-DTA analyses concerning the influence of recycled sand on the formation of hydration products and CH content. Figure 6a shows that the heat flux rate directly reflects the instantaneous reaction intensity of cement hydration, with significant differences in heat flux characteristics observed across different recycled sand groups. Mortar incorporating DLS exhibited the highest peak heat flow during the early stage (12–24 h), with the peak occurring slightly earlier than in other groups. It was the only group where the heat flow intensity was significantly higher than that of the control group. The fundamental reason lies in the double carbonate nature of its primary mineral, dolomite (CaMg(CO_3_)_2_), and calcite (CaCO_3_). On the one hand, as highly effective crystallisation nuclei for hydration products (C-S-H, Ca(OH)_2_), they significantly accelerate early-stage hydration (nucleation effect) [45]. On the other hand, the reaction between carbonates and C_3_A in the cement produces calcium aluminate hydrates, releasing additional heat. This dual effect drives both an increase in the peak heat flow and its earlier onset [46]. Mortar incorporating FS exhibited the second-highest thermal flow peak, slightly exceeding that of the control group. Its primary minerals are calcite (CaCO_3_) and microcrystalline quartz (SiO_2_). The nucleating effect of calcite slightly accelerates hydration, while the weak pozzolanic activity of microcrystalline quartz reacts slowly with Ca(OH)_2_ to form C-S-H gel and release a small amount of heat. Overall, its setting-promoting effect is weaker than that of DLS but stronger than that of inert river sand. River sand (reference) mortar exhibits a peak heat flow at the reference level, relying solely on the cement’s own hydration exothermic reaction without any additional acceleration or inhibition. This is primarily because river sand consists mainly of inert quartz (SiO_2_), which does not undergo chemical reactions with the cement paste but serves only as a physical filler. Mortar incorporating PFS exhibited the lowest peak heat flow, with the peak occurring significantly later (delayed by 8–10 h compared to the control group). Its early heat release rate was markedly lower than that of the other three groups. This occurs because PO_4_^3−^ ions leached from apatite (Ca_5_(PO_4_)_3_) adsorb onto the cement particle surface, forming a dense passivation film that impedes contact between cement particles and water. This significantly inhibits early hydration (retarding effect), resulting in a reduced and delayed peak heat release [30].

Figure 6b shows the cumulative heat map of the mortar, reflecting the total heat released during cement hydration over 0–72 h. This directly correlates with the degree of hydration, exhibiting a trend that is entirely consistent with the heat flow rate. The DLS mortar in the figure exhibits the highest cumulative heat release over 72 h, indicating the highest degree of hydration. This stems from the dual effects of nucleation and carbonaluminate reactions, which substantially increase the yield of hydration products, resulting in a significantly higher total heat release compared to other formulations. The cumulative heat release of FS mortar ranks second, slightly higher than that of the control group. This is attributable to the synergistic effect of mild acceleration and weak pozzolanic reaction, resulting in total heat release marginally exceeding that of inert river sand. The PFS mortar exhibited the lowest cumulative heat release over 72 h and the least degree of hydration. This was attributable to the retarding effect of PO_4_^3−^, which inhibited the full progression of the hydration reaction. Consequently, fewer hydration products were formed, resulting in a significantly lower total heat release compared to the other three groups. In summary, the differential effects of the three types of recycled sand on cement hydration heat fundamentally stem from variations in the interaction between their core minerals and the cement hydration system. Bicarbonate minerals (dolomite and calcite) accelerate setting through nucleation effects and additional chemical reactions. Calcite and weakly pozzolanic minerals provide mild setting acceleration, while apatite containing PO_4_^3−^ retards setting via passivation film effects.

### 3.6. Pore Structure of Mortar

**Figure 7:**
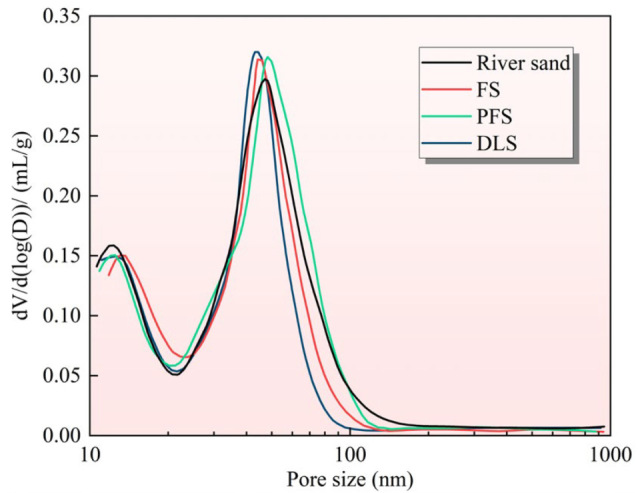
Effect of Different Recycled Sands on Mortar Porosity.

Pore structure (porosity, most probable pore size, and pore size distribution) constitutes the core microscopic factor determining mortar’s mechanical properties and water absorption behaviour. Figure 7 illustrates the influence patterns of different recycled sands on mortar pore structure after 28 d of curing. The reference group in the figure exhibits a porosity of 16.52%, with the three categories of recycled sand groups showing significant differentiation in porosity and most probable pore size. The porosity of FS, PFS, and DLS mortars was 15.43%, 18.26%, and 13.69%, respectively, with DLS reducing porosity by 17.13% and PFS increasing it by 10.53%. Among these, DLS mortar exhibits the smallest maximum pore size, while PFS mortar displays the largest maximum pore size, with a difference exceeding 40%. The porosity of PFS mortar is the highest, with the largest pore size distribution, a phenomenon directly attributable to its hydration-inhibiting properties. This is partly due to the dissolution of apatite, forming a passivation film, which significantly inhibits the early hydration of cement. Even after 28 d of curing, the degree of hydration remains lower than that of other groups. Insufficient formation of hydration products such as C-S-H gel and calcium hydroxide results in inadequate filling of the primary pores within the paste matrix, leading to a significant increase in the proportion of large-aperture pores [47]. On the other hand, apatite lacks nucleation effects and pozzolanic activity, and cannot generate additional hydration products through secondary reactions to fill pores [48]. The pore structure exhibited the weakest optimisation capability, ultimately resulting in the highest porosity and largest pore size.

The FS mortar demonstrated lower porosity than the control group, with the smallest possible pore size being smaller than that of the control group, indicating a mild degree of pore structure optimisation. This is closely related to its hydration characteristics. The calcite in FS exhibits a nucleation effect, accelerating cement hydration and promoting more complete C-S-H gel formation. This facilitates earlier filling of capillary pores within the paste, thereby reducing the proportion of macropores. Moreover, calcite exhibits superior compatibility with cement paste compared to quartz, resulting in a denser interfacial transition zone that minimises the formation of large pores at the interface. Consequently, the resulting pore structure proves superior to that of the control group. DLS mortar exhibits the lowest porosity and smallest mean pore size, representing the group with the most pronounced pore structure optimisation effect, primarily due to its strong hydration-promoting properties. On the one hand, the double carbonate minerals of dolomite and calcite exhibit a pronounced nucleation effect, significantly enhancing the degree of hydration. This results in the highest production of C-S-H gel and the most thorough filling of slurry pores [49]. On the other hand, carbonates react with C_3_A in the cement to form calcium aluminate hydrates, which further fill the pores within the paste and at interfaces, refining the pore size distribution [50]. The above analysis demonstrates a significant negative correlation between the degree of hydration and pore structure. More complete hydration (DLS > FS > river sand > PFS) results in more thorough C-S-H gel filling, smaller mean pore diameters, and lower porosity. The core influencing factor is aggregate reactivity/nucleation effect. The nucleation effect of carbonate aggregates (dolomite, calcite) can significantly refine pore structure, whereas inert quartz offers no optimisation effect; conversely, the retarding action of apatite deteriorates pore structure. This principle directly accounts for the macroscopic performance differences: the DLS and FS groups exhibit superior mechanical properties and lower water absorption due to pore refinement and reduced porosity. The PFS group, characterised by high porosity and large pore size, exhibits limited enhancement in early mechanical properties and higher water absorption rates, thereby forming a logical closed-loop relationship between microstructure and macroscopic performance.

**Figure 8:**
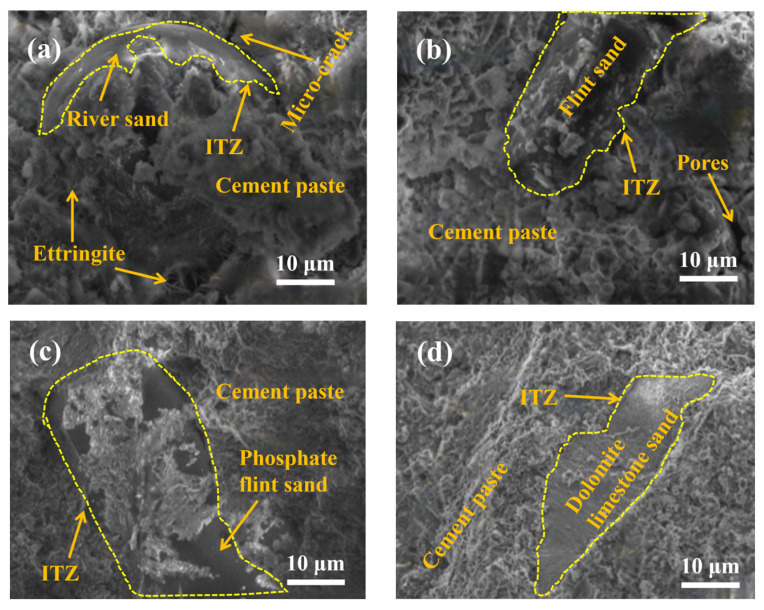
SEM images of river sand mortar and mortars with different recycled sands: (**a**) River sand; (**b**) FS; (**c**) PFS; (**d**) DLS.

Figure 8 illustrates the microstructural differences between river sand mortar and three types of recycled sand mortar after 28 d of curing. Figure 8a demonstrates that a distinct interfacial gap exists between river sand particles and the cement paste matrix, with numerous pores and microcracks distributed throughout the paste. The interfacial bonding is markedly loose. This phenomenon stems from the physical properties of river sand. River sand possesses a smooth surface and a low water absorption rate (merely 2.76%). During mixing, a continuous water film readily forms on the particle surfaces. Upon evaporation of this film, voids remain between the aggregate and paste, resulting in a loose interface transition zone (ITZ) [51]. Moreover, river sand consists of inert quartz particles lacking chemical reactivity, which merely bind to the slurry through physical filling, resulting in weak cohesive strength. Concurrently, the highly mobile ions forming calcium aluminate cement in the solution permeate into pores and cracks where they crystallise, forming “bridging calcium aluminate cement” [52]. Although partially filling defects, it cannot fundamentally improve the quality of the interface bonding. This also explains why the flexural strength and compressive strength of the reference group are lower than those of most recycled sand groups. Figure 8b indicates that the interfacial tension between FS particles and the cement paste is tighter than that of the reference group, with a significant reduction in interfacial gaps. However, a small number of dispersed air voids and microcracks remain within the paste, resulting in bonding performance that is slightly inferior to the other two types of recycled sand. The primary reason lies in the FS’s surface roughness and distinctive elongated flake structure, which enhances bonding with the paste through mechanical interlocking forces. Furthermore, its higher water absorption rate (5.78%) compared to river sand enables adsorption of free water, thereby promoting interfacial hydration and resulting in a denser interfacial transition zone (ITZ). However, the slender, flake-like particles tend to interlock and entangle, hindering air release within the paste during mixing and leading to localised porosity defects. Consequently, their interface integrity is slightly inferior to that of PFS and DLS. Figure 8c,d demonstrate that the apatite rock and dolomite limestone particles are in complete and intimate contact with the cement paste, with virtually no discernible interfacial gaps. The paste structure is uniformly dense, exhibiting no significant pores or cracks, representing the group with the highest interfacial bonding quality among the three types of recycled sand. Moreover, its water absorption rate is significantly higher than that of river sand (6.38% for PFS, 7.10% for DLS). During mixing, it adsorbs free water within the slurry, reducing the formation of an interfacial water film. Subsequently, it slowly releases moisture, providing sustained hydration conditions for unhydrated cement particles at the interface. This promotes the extensive formation of hydration products such as C-S-H gel and calcium aluminate hydrate at the interface, thereby filling minute voids. Moreover, the double carbonate nucleation effect of DLS and the particle-filling effect of apatite rock further promote the dense accumulation of interfacial hydration products (consistent with the “minimum pore size” observed in pore structure analysis), ultimately forming a microstructure where the aggregate, hydration products, and paste are tightly interconnected. This significantly enhances the interfacial bonding strength.

The SEM analysis results clearly corroborate the logical relationship between microstructure and macroscopic properties. The closer the interface bonding (DLS, PFS > FS > river sand), the superior the mortar’s mechanical properties (higher compressive and flexural strength) and the weaker its water absorption (lower water absorption rate). This trend is entirely consistent with the previously described variations in mechanical properties and water absorption. Moreover, the surface roughness and high water absorption of recycled sand are pivotal factors in enhancing interfacial bonding. Particle morphology (such as the flake-like structure of FS) may introduce localised defects, necessitating mitigation through optimised replacement ratios (e.g., the optimal proportion of 25%).

### 3.7. Heavy Metal Ion Concentrations in Mortar

Heavy metal leaching toxicity serves as a core indicator for assessing the environmental safety of recycled building materials. It is generally recognised that the higher the proportion of recycled sand incorporated, the greater the potential risk of heavy metal leaching. Therefore, this study selected mortar specimens with 100% recycled sand replacement and cured for 28 d for leaching tests. The concentrations of eight heavy metal ions, including Ag, As, and Ba, in the leachate were determined via ICP-OES (Table 4), aiming to verify the environmental compatibility of phosphorite waste rock recycled sand mortar. The heavy metal leaching concentrations of the reference group mortar (100% river sand) in the table serve as the benchmark. The leaching patterns of the three recycled sand groups exhibit significant differentiation. The leaching concentrations of Ag (0.012 mg/L), As (0.022 mg/L), and Cr (0.120 mg/L) in FS mortar exceeded those of the control group (0.002 mg/L, 0.010 mg/L, and 0.090 mg/L, respectively), while concentrations of all other elements were consistent with the control group. The PFS mortar group exhibited only a slightly higher Cr leaching concentration (0.114 mg/L) compared to the control group, with all other element concentrations being equivalent to or lower than those of the control group. The leaching concentrations of all heavy metal elements in DLS mortar were lower than those in the control group. Specifically, arsenic concentrations decreased from 0.010 mg/L to 0.002 mg/L, while chromium concentrations fell from 0.090 mg/L to 0.062 mg/L, demonstrating the most effective heavy metal immobilisation performance. The data above indicate that the leaching concentrations of Ba in all three types of recycled sand mortar were lower than those of the control group (control group: 0.350 mg/L; recycled sand groups: 0.112–0.325 mg/L). The leaching concentrations of Cd, Hg, Pb, and Zn remained stable at 0.002 mg/L, identical to the control group, with no significant fluctuations observed. Although the leaching concentrations of certain heavy metals in some recycled sand groups were marginally higher than those in the control group, all elements remained well below the maximum permissible concentrations stipulated in GB 5085.3-2007 [53] (Cr ≤ 1.5 mg/L, As ≤ 1.5 mg/L, Pb ≤ 3.0 mg/L). This confirms the sound environmental safety of phosphorite waste rock and recycled sand mortar.

The primary reason for the low heavy metal leaching concentrations is closely related to the microstructure of the mortar and the characteristics of the recycled sand. The analysis identifies three primary reasons: First, the dense microstructure formed by recycled sand mortar (e.g., the DLS group exhibits a porosity of merely 13.69% with tightly bonded interfaces) substantially reduces migration pathways for heavy metal ions, thereby impeding their diffusion into the leachate. Second, recycled sand possesses inherent high hardness and forms a robust interface transition zone (ITZ) with the cement paste, physically encapsulating heavy metal ions within the hydration product network and thereby reducing leaching potential. Third, the C-S-H gel and Ca(OH)_2_ produced during cement hydration possess the capacity to adsorb heavy metal ions, thereby further immobilising certain heavy metals and reducing the risk of leaching. In summary, although mortar prepared using recycled sand from phosphate rock waste rock exhibits differences in the leaching concentrations of certain heavy metal elements compared to mortar made with river sand, both meet national environmental protection standards and present controllable environmental safety. This outcome provides crucial environmental support for the engineering application of recycled sand from phosphate mine tailings, while also validating the intrinsic logic of recycled sand mortar: “microstructural compaction-controlled heavy metal leaching”.

### 3.8. Cost–Benefit Analysis of Recycled Sand from Phosphate Mine Waste Rock

Cost-effectiveness is a core consideration in the engineering application of recycled building materials. This study calculates the preparation cost per cubic metre of mortar and the “standardised cost per 1 MPa increase in flexural strength” based on the optimal flexural strength replacement ratios for different recycled sands (FS 75%, PFS 50%, DLS 25%). The aim was to quantify the economic advantages of recycled sand, with the results shown in Table 5. Cost calculations focus on variations in sand material costs, with commercially available river sand priced at CNY 0.106 per kilogram. Recycled sand is derived from the processing of waste rock from phosphate mines, eliminating the need for additional procurement. Only the basic costs of crushing and screening are incurred (which are significantly lower than the purchase price of river sand and can be considered negligible). Consequently, the higher the proportion of recycled sand used as a replacement, the more pronounced the savings in sand material costs become. Cost calculations are based on the unit of “cubic metre of mortar”, with the core reference being the variation in river sand usage across each group (Table 5). The replacement rate of recycled sand is calculated as: (1−River sand usage/1171.88 kg) × 100%, directly reflecting the extent of savings in sand material costs.

A comparison of unit costs between the benchmark group (100% river sand) and the recycled sand group reveals that the benchmark group (100% river sand) incurs a cost of CNY 124.22 per cubic metre, relying entirely on commercially sourced river sand. The cost of recycled sand mixtures showed a significant reduction: the 25% river sand + 75% FS mixture had the lowest cost (CNY 30.95/m^3^), saving CNY 93.27/m^3^ compared to the baseline mixture, representing a reduction of 75.1%. 50% river sand + 50% PFS mixture composition: CNY 62.11/m^3^, saving CNY 62.11/m^3^; 75% river sand + 25% DLS aggregate composition: CNY 93.06 per cubic metre, saving CNY 31.16 per cubic metre. The core reason for cost savings lies in the substitution of high-priced river sand with recycled sand, with greater substitution rates yielding more pronounced savings (e.g., the maximum savings correspond to a 75% replacement rate). This is directly attributable to recycled sand’s resource recovery properties, transforming waste into valuable resources, while simultaneously reducing environmental management costs associated with phosphorite waste rock stockpiling.

To more objectively evaluate the cost-performance ratio, the concept of a strength standard value (CNY/m^3^/MPa), denoting the cost per unit of flexural strength, has been introduced. The results indicate: Strength-standardised cost ranking: 25% river sand + 75% FS (CNY 4.18/m^3^/MPa) < 50% river sand + 50% PFS rock (CNY 8.51/m^3^/MPa) < 75% river sand + 25% DLS (CNY 13.11/m^3^/MPa) < 100% River sand (CNY 18.82/m^3^/MPa). Data indicate that the recycled sand group not only exhibits lower direct costs but also demonstrates significantly reduced input costs per unit strength compared to the benchmark group. For instance, the 25% river sand + 75% FS group achieved a flexural strength of 7.4 MPa (a 12.1% improvement over the control group), yet its unit strength cost amounted to merely 22.2% of that of the control group, delivering the dual advantage of enhanced performance coupled with reduced costs. Even the DLS group, featuring a lower replacement ratio, demonstrated superior cost-effectiveness compared to the pure river sand group.

The above analysis demonstrates that the FS group not only exhibits superior late-stage flexural strength (7.39 MPa) but also achieves maximum cost savings through its high replacement ratio (75%), thereby representing the optimal combination of performance and cost. When the PFS group (50% replacement ratio) achieved a flexural strength of 7.29 MPa (an increase of 12.12%), its cost and cost-effectiveness were both at a moderate level, making it suitable for scenarios demanding high strength stability. The DLS group (25% replacement ratio) demonstrated a significant advantage in early flexural strength (5.59 MPa). Although the low replacement ratio resulted in modest cost savings, its cost-effectiveness remained superior to the control group, making it suitable for projects requiring early-stage performance. In summary, utilising recycled sand from phosphate mine tailings as a substitute for river sand in mortar preparation not only enhances mechanical properties, improves microstructure, and alleviates environmental pressures, but also achieves significant cost savings by reducing river sand procurement. Furthermore, it offers a more advantageous cost per unit strength. This synergy between economic characteristics and technical performance provides robust support for the engineering application of recycled sand, achieving a true integration of ecological, economic, and technical benefits.

## 4. Conclusions

This study systematically investigated the feasibility of using recycled sand from three types of phosphate mine waste rock—flint (FS), phosphorite flint (PFS), and dolomitic limestone (DLS) to replace river sand in mortar preparation. Through comprehensive analysis of macro-performance, microstructure, hydration characteristics, environmental safety, and cost-effectiveness, the following core conclusions were drawn.

(1) All three types of recycled sand can serve as substitutes for river sand in construction applications without adversely affecting the hydration process of mortar. XRD and TG-DTA analyses confirm that recycled sand retains its original mineral composition while promoting complete cement hydration to form key hydration products such as Ca(OH)_2_ and C-S-H gel. No unhydrated cement clinker residues are present, verifying its low reactivity and compatibility.

(2) The high water absorption rate of recycled sand and its rough surface cause the slump to decrease continuously with increasing replacement ratio, with FS exhibiting the most pronounced effect. Setting time shortens with increasing replacement ratio, with DLS exhibiting the most pronounced acceleration effect. The water absorption rate exhibits a trend of initially decreasing and then increasing with rising recycled sand replacement levels. At a 25% replacement rate, the water absorption rate reaches its lowest point, with the DLS group showing the most significant reduction (47.69%). At higher replacement levels, the formation of interconnected pores due to particle interlocking leads to an increase in water absorption. In terms of mechanical properties, DLS facilitates earlier enhancement (21.52% increase in 7 d strength at 25% replacement), while FS (75% replacement) and PFS (50% replacement) demonstrate more pronounced late-stage reinforcement (11.97–12.12% increase). The optimum replacement ratio for compressive strength was uniformly 25%, with PFS demonstrating the most pronounced enhancement effect (a 10.91% increase in 28 d strength). Higher replacement ratios resulted in strength reduction due to structural defects.

(3) The heavy metal leaching concentrations of all three types of recycled sand mortar were significantly below national limits, with all heavy metal contents in the DLS group lower than those in the river sand group, demonstrating excellent environmental compatibility. Cost analysis indicates that recycled sand can substantially reduce mortar preparation costs. The 25% river sand + 75% FS mixture offers the most favourable cost-performance ratio, with a unit strength cost amounting to merely 22.2% of that for pure river sand. This yields savings of CNY 93.27 per cubic metre.

(4) DLS recycled sand is suitable for projects requiring high early strength and impermeability (25% replacement ratio); FS recycled sand is suitable for applications requiring post-curing strength and cost control (75% replacement rate); and PFS recycled sand is suitable for projects demanding high strength stability (50% replacement rate). The use of recycled sand from phosphorite waste rock to replace river sand in mortar production achieves multiple objectives: resource recovery from solid waste, resource conservation, environmental burden reduction, and cost minimisation. This approach provides technical support for the high-value utilisation of mining waste, demonstrating significant engineering application value and ecological-economic benefits.

## Figures and Tables

**Table 1:** Chemical constituents of three kinds of limestones (Mass fraction/%).

	CaO	SiO_2_	MgO	Al_2_O_3_	Fe_2_O_3_	Na_2_O	K_2_O	P_2_O_5_	TiO_2_	LOI
FS	52.57	4.35	0.88	0.36	0.13	0.23	0.36	0.19	0.21	40.72
PFS	51.48	3.32	0.64	0.45	0.19	0.36	0.40	0.74	0.26	42.16
DLS	51.09	4.33	0.91	0.43	0.22	0.22	0.38	0.21	0.28	41.93

**Table 2:** Physical Properties of Sand.

Varieties	Specific Gravity (g/cm^3^)	Sand Equivalent (%)	Fine Sand Content% (<63 μm)	Fineness Modulus	Water Absorption Rate (%)	pH
River sand	2.72	66.10	10.30	3.11	2.76	8.52
FS	2.61	73.82	7.07	3.20	5.78	8.35
PFS	2.67	67.50	11.00	2.95	6.38	8.45
DLS	2.76	42.10	26.85	3.02	7.10	9.17

**Table 3:** Chemical composition of cement.

Composition	CaO	SiO_2_	Al_2_O_3_	Fe_2_O_3_	MgO	f-CaO	SO_3_	LOI
Mass fraction/%	64.32	20.22	4.81	3.14	3.31	0.79	1.29	2.12

**Table 4:** Heavy Metal Ion Concentrations in Mortar at 100% Recycled Sand Replacement.

Element	Concentration (mg/L)			
	River Sand	FS	PFS	DLS
Ag	0.002	0.012	0.002	0.002
As	0.010	0.022	0.010	0.002
Ba	0.350	0.112	0.262	0.325
Cd	0.002	0.002	0.002	0.002
Cr	0.090	0.120	0.114	0.062
Hg	0.002	0.002	0.002	0.002
Pb	0.002	0.002	0.002	0.002
Zn	0.002	0.002	0.002	0.002

**Table 5:** Cost Estimate for Mortar Prepared Using Different Aggregates.

Group	100% River Sand	25% River Sand + 75%FS	50% River Sand + 50%PFS	75% River Sand + 25%DLS
River sand consumption (kg/m^3^)	1171.88	291.97	585.94	877.91
Cost (CNY/m^3^)	124.22	30.95	62.11	93.06
28 d Flexural strength (MPa)	6.6	7.4	7.3	7.1
Standard value of strength (CNY/m^3^/MPa)	18.82	4.18	8.51	13.11

## Data Availability

The original contributions presented in this study are included in the article. Further inquiries can be directed to the corresponding author.
